# Progressive Subvalvular Right Ventricular Outflow Tract Obstruction With a Single Coronary Artery in an Adult

**DOI:** 10.1016/j.jaccas.2026.107522

**Published:** 2026-03-20

**Authors:** Masashi Koga, Rei Ito, Syoko Doi, Atsuhiko Sugimoto

**Affiliations:** a Department of Cardiology, Sagamihara Kyodo Hospital, Sagamihara, Japan; b Department of Cardiology, St. Marianna University School of Medicine, Kawasaki, Japan

**Keywords:** multimodality imaging, right ventricular outflow tract obstruction, single coronary artery

## Abstract

**Case Summary:**

A 57-year-old man with a history of ventricular septal defect and prior infective endocarditis presented with progressive exertional dyspnea. Multimodality imaging demonstrated severe subvalvular right ventricular outflow tract (RVOT) obstruction with a marked intraventricular pressure gradient (190 mm Hg proximal right ventricle vs 15 mm Hg pulmonary artery). Coronary angiography revealed a single coronary artery arising from the left coronary sinus (Lipton type L1), with a branch crossing anterior to the RVOT.

**Discussion:**

Progression of obstruction suggested evolving double-chambered right ventricular physiology and/or superimposed infection-related remodeling. The anomalous coronary course had important procedural implications.

**Take-Home Messages:**

Progressive subvalvular RVOT obstruction in adults warrants consideration of both congenital mechanisms, such as double-chambered right ventricle, and acquired causes including prior infective endocarditis. Comprehensive multimodality imaging is essential to define coronary anatomy and hemodynamic severity before considering intervention.

## Graphical Abstract

**Figure:**
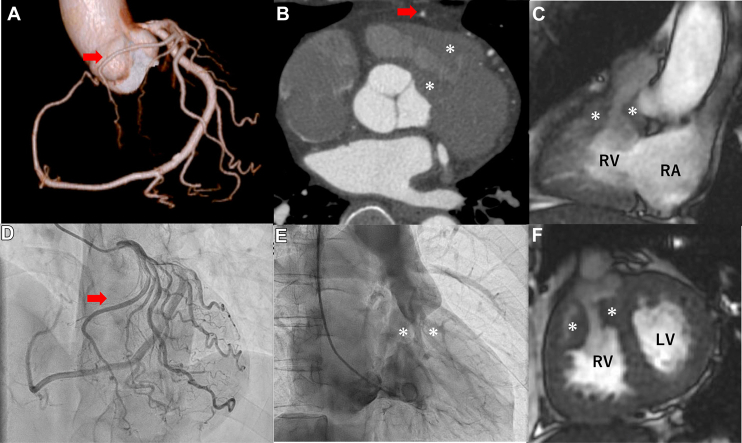

A 57-year-old man was referred to our hospital for evaluation of cardiac murmur and progressive exertional dyspnea. He had been diagnosed with a ventricular septal defect (VSD) at birth and was followed regularly until adolescence. Eight years earlier, he was hospitalized for infective endocarditis involving a vegetation located in the subpulmonary region of the right ventricular outflow tract (RVOT) at another institution. He was successfully treated with intravenous antibiotics. At that time, transthoracic echocardiography demonstrated a subvalvular RVOT pressure gradient of 54 mm Hg, and double-chambered right ventricle (DCRV) was suspected. The VSD was not clearly visualized during that admission. Two years before presentation, cardiac computed tomography suggested a single coronary artery (Figures 1A and 1B, Video 1), and he remained asymptomatic under conservative follow-up.

**Figure 1:**
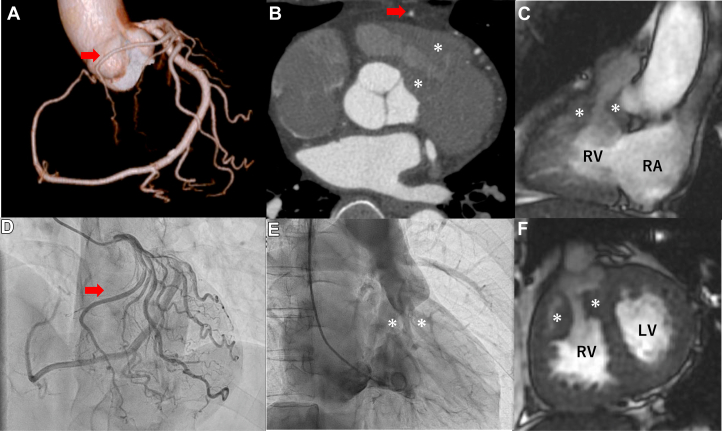
Multimodality Imaging of RVOT Obstruction and Single Coronary Artery (A and B) Cardiac computed tomography showed a single coronary artery arising from the left coronary sinus (Lipton type L1). A branch of the left anterior descending artery courses anteriorly across the right ventricular outflow tract (red arrow). (C and F) Cardiac magnetic resonance demonstrating focal Right Ventricular Outflow Tract (RVOT) narrowing without residual ventricular septal defect (asterisks). (D) Coronary angiography revealing a single coronary artery arising from the left coronary sinus (Lipton type L1). A branch of the left anterior descending artery courses anteriorly across the right ventricular outflow tract (red arrow). (E) Right ventriculography demonstrating severe RVOT obstruction with a marked pressure gradient (asterisks). The asterisks indicate the focal subvalvular narrowing of the right ventricular outflow tract (RVOT). LV = left ventricle; RA = right atrium; RV = right ventricle.

On admission, electrocardiography demonstrated right ventricular hypertrophy. Transthoracic echocardiography revealed severe RVOT obstruction with a peak velocity of 5.0 m/s. Cardiac magnetic resonance demonstrated focal subvalvular narrowing without evidence of a residual VSD (Figures 1C and 1F). Coronary angiography demonstrated a single coronary artery arising from the left coronary sinus. The left circumflex artery continued in the atrioventricular groove and supplied the right coronary artery territory, consistent with Lipton classification type L1 (Figure 1D).^1^ A branch of the left anterior descending artery was observed to course anteriorly and cross the RVOT, indicating a potentially hazardous anatomy for surgical intervention. Right heart catheterization demonstrated a markedly elevated proximal right ventricular systolic pressure of 190 mm Hg. Right ventriculography revealed focal subvalvular narrowing (Figure 1E). Distal to the obstruction, pulmonary artery systolic pressure was approximately 15 mm Hg, resulting in a peak-to-peak gradient of 175 mm Hg, confirming severe hemodynamic obstruction. Given the presence of symptoms and severe focal subvalvular RVOT obstruction, surgical intervention was recommended. However, after detailed discussion regarding procedural risks, the patient declined surgery. The patient has been managed with close clinical and imaging follow-up.

In adults, DCRV is a well-recognized cause of focal subvalvular RVOT obstruction and is frequently associated with VSD.^2^ In this case, a prior VSD diagnosis and documented moderate RVOT gradient support a congenital substrate. The gradient progressed from 54 mm Hg 8 years earlier to 175 mm Hg at present, confirming marked hemodynamic progression. Although this progression strongly supports an underlying congenital mechanism, prior infective endocarditis involving the subpulmonary RVOT region may also have contributed to localized fibrosis and exacerbated obstruction. Thus, both congenital DCRV and superimposed infection-related structural changes may have played a role in the present severe obstruction. The presence of a single coronary artery with a branch of the left anterior descending artery crossing anterior to the RVOT has important implications for surgical planning. Although not an interarterial malignant course, this anatomy increases the risk of coronary injury during surgical interventions.

This case highlights that progressive subvalvular RVOT obstruction in adults may reflect combined congenital and acquired mechanisms. Comprehensive multimodality imaging, including careful delineation of coronary anatomy, is essential to guide safe therapeutic decision-making.

## Funding Support and Author Disclosures

The authors have reported that they have no relationships relevant to the contents of this paper to disclose.

## Appendix

Video 1 Cardiac Computed Tomography Demonstrating a Single Coronary Artery Arising From the Left Sinus of Valsalva (Lipton L1) The left circumflex artery continues to supply the right coronary territory. A branch of the left anterior descending artery is seen crossing anterior to the right ventricular outflow tract.
